# Integrating routinely collected mosquito vector and human behavioral data for malaria prevention: A case example in central region Malawi

**DOI:** 10.1186/s12936-026-06043-3

**Published:** 2026-07-15

**Authors:** Kaci D. McCoy, Allison Hendershot, Abdoulaye Bangoura, Leonard Dandalo, Michael Bride, Shadreck Mulenga, Akuzike Banda, John Munthali, Themba Mzilahowa, Sophia Kaufman, Stella Babalola, Shelby Cash, John E. Gimnig, Gabrielle Hunter, Bolanle Olapeju, Joseph Millward, Neil F. Lobo, Sarah Zohdy, Jenny Carlson Donnelly, April Monroe

**Affiliations:** 1 Breakthrough ACTION Project, Johns Hopkins Center for Communication Programs, Baltimore, MD USA; 2 PMI VectorLink Project, Abt Associates, Rockville, MD USA; 3 PMI VectorLink Project, Abt Associates, Lilongwe, Malawi; 4 U.S. President’s Malaria Initiative, Centers for Disease Control and Prevention, Atlanta, GA USA; 5 Uniformed Services University of the Health Sciences, Bethesda, MD USA; 6 Eck Institute for Global Health, University of Notre Dame, Notre Dame, IN USA; 7 U.S. President’s Malaria Initiative, United States Agency for International Development, Washington D.C., USA; 8 National Malaria Control Program, Ministry of Health, Lilongwe, Malawi; 9 Malaria Alert Centre, Kamuzu University of Health Sciences, Blantyre, Malawi

**Keywords:** Vector-human interaction, Residual malaria transmission, Insecticide-treated nets, Malaria

## Abstract

**Background:**

Integrating human behavioral data with data on malaria vector biting rates can help to quantify human exposure to malaria vectors and identify gaps in protection; however, to date, the availability of necessary human location and behavior data have been more limited as compared to that of corresponding entomological data. To expand the data available for use in vector-human data integrations, this case example explored the process and interpretations of integrating human behavior estimates from the Malaria Behavior Survey (MBS), a large-scale cross-sectional survey measuring behaviors and their determinants, with routinely collected VectorLink entomological surveillance data in Central Malawi.

**Methods:**

This study followed established guidance on vector-human data integration to guide extraction of necessary data inputs from existing, routinely collected data. Hourly nighttime human behavior and location estimates, as well as population insecticide treated bednet (ITN) use rate, were derived from questions in the Malawi MBS, conducted in Malawi in May–July 2021. Hourly nighttime human biting rates were derived from human landing catches (HLCs) carried out by VectorLink in Malawi in June 2021. Human and vector data were then integrated to provide weighted estimates of exposure to vector bites, accounting for proportions of people indoors or outdoors, and protected by ITNs, each hour of the night.

**Results:**

When accounting for mosquito and human location, most exposure to vector bites was estimated to occur indoors (96%), at times when people were asleep (87%). Although most mosquito bites occurred indoors during sleeping hours—when ITNs could offer protection—only 35% of all exposure to bites was estimated to be prevented, considering that the population-level usage rate of ITNs was only 40%. ITN access, which was only 39%, was identified as an important factor contributing to the protection gap.

**Conclusion:**

This case example suggests that integrating routinely collected entomological and human behavioral data can provide programmatically relevant insights. Future studies or programs may consider data integration of similar sources to better inform vector control or social and behavior change activities.

**Supplementary Information:**

The online version contains supplementary material available at https://doi.org/10.1186/s12936-026-06043-3.

## Background

The incidence of malaria declined by 37% worldwide from 2000 to 2015, due in part to the large-scale implementation of effective vector control interventions [1], primarily insecticide-treated bed nets (ITNs) and indoor residual spraying (IRS). However, since 2019, progress towards reducing the malaria burden has stalled for many countries [2–5]. To be effective, malaria vector control relies on susceptible malaria vectors overlapping with an intervention’s effective space. For ITNs, which have been a cornerstone of malaria prevention across sub-Saharan Africa, this is indoors, at night, when the ITN is in use [6, 7]. The rise of insecticide resistance [8, 9] and outdoor vector biting [10], shifts in species composition [11], the emergence of secondary vector species [12–14], and vector behavioral adaptations to circumvent insecticide exposure [15, 16] have arisen as challenges to ITN effectiveness in various contexts across sub-Saharan Africa. For example, vector biting in the early evening and late morning hours has been seen for both primary and secondary malaria vector species in some contexts, which corresponds with times when many people are awake or away from the house, i.e., unprotected by ITNs [12, 13, 17, 18].

The protection conferred by ITNs is also driven by timing and levels of ITN use in a population. ITN access is a key driver of ITN use, and it is important to measure and distinguish between gaps in use driven by insufficient access versus other barriers to use, like those related to ITN preference, norms, attitudes, or perceived efficacy, as the underlying challenge can point to different types of solutions [19]. Sub-optimal access, for example, may suggest the need to increase the supply of nets through appropriate channels, determine different channels for distribution, and/or to address issues with chemical and physical ITN durability. Gaps in use of available nets may suggest the need to understand and address context-specific individual, social, or environmental barriers to ITN use.

As challenges in malaria vector control arise, context-specific evidence is critical to understanding what gaps in protection [7] exist in a given environment and how to respond. Integrating human and mosquito behavior data may help identify gaps in protection related to access to ITNs, use of available ITNs, how well ITNs work while in use, and exposure that can occur outside of the times and places that ITNs provide protection. Identification of these gaps can inform where and how to tailor vector control interventions for malaria.

Previous studies have demonstrated how integrating human behavioral data with malaria vector biting rate data can improve estimates of patterns of human exposure to malaria vectors within a given context and identify gaps in ITN protection [18, 20–23]. Despite the utility of combining these measures, data on nighttime human behavior patterns have been relatively limited in comparison to data on entomological patterns; previous studies investigating transmission dynamics have relied on human behavior data from a limited number of studies [24]. While there is no standard approach to estimate nighttime human behavior patterns, the use of survey questions to approximate an individual’s location throughout the night is well established [18, 20, 25, 26]. Importantly, a recent study demonstrated the utility of incorporating questions to estimate human behavior in large-scale surveys, like the Malaria Indicator Survey (MIS), which can provide robust, longitudinal data for such integrations [18].

This article presents how existing human malaria-related behavior and location data from the Malaria Behavior Survey (MBS)—a standardized cross-sectional household and individual survey designed to measure malaria-related behaviors and their underlying determinants [25]—can be integrated with routine entomological monitoring data collected through the PMI VectorLink and Evolve projects to estimate context-specific human exposure to malaria vectors, thereby expanding the available human behavior data for these analyses. Leveraging these existing data sources may support data-driven decision making in resource-limited contexts. Emphasis is given to the process, interpretation, and lessons learned to provide guidance to programs that may wish to use these methods in the future.

## Methods

### Study area

This study was conducted using human and vector data collected within the Central region of Malawi. The Central region of Malawi was chosen as malaria remains a major health burden for this area, with the 2021 Malawi MIS estimating a parasite prevalence of 12% for children under five [26], and the region had complete human and entomological data sets with strong geographic and temporal overlap. The Malawi MBS data collection took place throughout the country, was fielded in May through July 2021, and was powered to make estimations of malaria-related behaviors at the national and survey zonal levels. The survey, which consisted of both a household survey and individual surveys for adult men and women, was conducted by the Breakthrough ACTION project in Malawi in collaboration with the National Malaria Control Program (NMCP), the U.S. President’s Malaria Initiative, and the local organization Imani Consultants Ltd. [25]. Detailed information on MBS methods and the full Malawi MBS report are available at https://malariabehaviorsurvey.org.

**Fig. 1:**
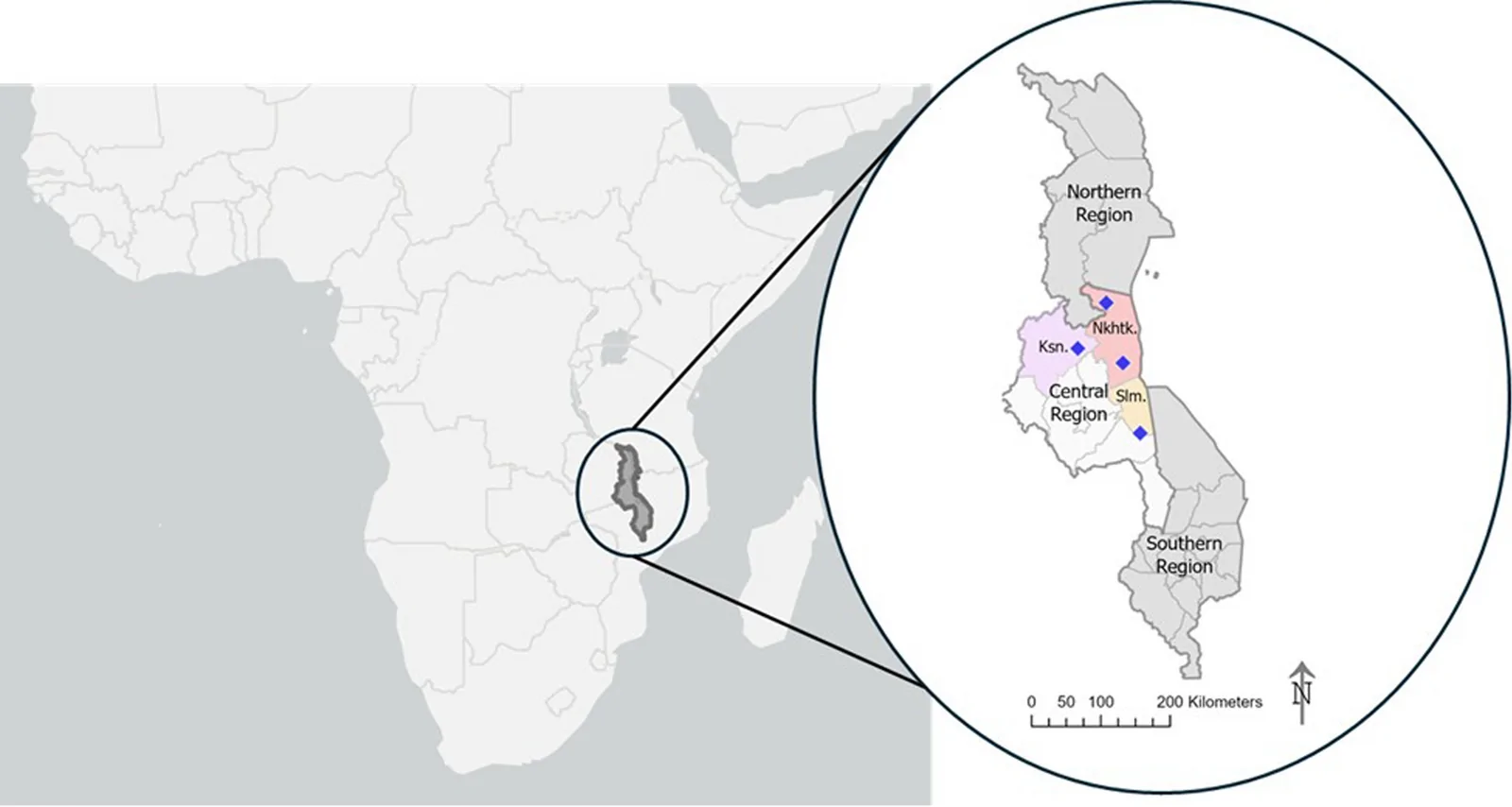
Study area selected for data integration. Entomological monitoring sites, identified with blue diamonds, fell within the three districts highlighted in the Central region (Nkhotakota (Nkhtk.), Kasungu (Ksn.), and Salima (Slm.)); the MBS was conducted throughout the Central region

Entomological monitoring was conducted by the VectorLink project in Malawi in collaboration with the Malaria Alert Centre (MAC) of Kamuzu University of Health Sciences in four sites/villages in three districts: Vwawa (S 12° 24′ 54′′; E 34° 5′ 9′) and Ngalauka (S 13° 10′ 38.52"; E 34° 18′ 12.8") in Nkhotakota district; Kachokolo (S 12° 59′ 35.09"; E 33° 43′ 4.19") in Kasungu district; and Chilungo (S 14° 3′ 46"; E 34° 31′ 40") in Salima district. These villages have been involved in routine entomological monitoring studies since 2019 [27]. These study sites were selected for the data integration as they fall within the Central zone (Fig. 1), and they had performed indoor and outdoor human landing catches (HLCs) during the same time frame as the MBS survey, i.e., in June 2021.

The study area experiences a tropical climate with two main seasons: a hot, rainy season from November to April, and a cool, dry season from May to October [28]. Climate data captured during entomological data collection, which occurred just after the end of the rainy season, indicated that the temperatures ranged from 11.7 °C–23.7 °C indoors and 9.5 °C–21.6 °C outdoors. Residents of these districts cultivate maize, cassava, and rice as the main staple food crops. Sugarcane, tobacco, and rice are also cultivated as cash crops. Fishing is the other primary economic activity in the three districts. Most houses in these villages are traditional African grass-thatched houses although many people also live in houses with corrugated iron roofs. The primary malaria vectors in this area are *An. funestus* s.s., *An. arabiensis* and *An. gambiae* s.s., and secondary vectors include *An. coustani, An. tenebrosus, An. pharoensis, An. maculipalpis, An. pretoriensis, An. rufipes, and An. squamosus* [30, 31]. In Malawi, malaria transmission is perennial and the entire population is at risk, but the disease incidence increases during the rainy season (November–April) especially along the lake shore and the central plains [5, 29, 30].

Malawi has implemented either IRS or ITNs for malaria vector control, depending on the district. ITNs are distributed via periodic mass distribution campaigns for universal coverage every three years, supplemented by routine distribution at antenatal care (ANC) and in labor and delivery wards at newborn discharge. The mass distribution campaign carried out prior to the data collection period occurred in September to December 2018, during which Kasungu district received ITNs treated with pyrethroids and Salima district received ITNs treated with pyrethroid + PBO; Nkhotakota district received IRS treatment only, although routine distribution through ANC and labor and delivery still occurred [2, 31]. The most recent IRS treatment prior to the data collection period occurred from October to December 2020, during which houses in Nkhotakota district were sprayed with either SumiShield 50WG (clothianidin at a target dose of 300 mg/m2) or Actellic 300CS (pirimiphos-methyl at a target dose of 1.0 mg/m2). SumiShield 50WG was sprayed in both sentinel sites in Nkhotakota district. Household spray coverage was 91% across the entire district [32].

### Study design

Indicators were calculated using the methods described by Monroe et al., which require four main inputs: 1. hourly nighttime indoor and outdoor human biting rates; 2. hourly nighttime indoor and outdoor human location estimates; 3. reported proportion of the human population that used an ITN while asleep; and 4. personal protection provided by an ITN while in use [33]. Vector-human analyses and resulting figures were generated in Microsoft Excel, version 2302, using the template created by Monroe et al. [34]. The template used for analysis is available in the supplemental materials (see Additional File 1). Resulting estimates for exposure are interpreted comparatively, not absolutely.

### Data sources

**Fig. 2:**
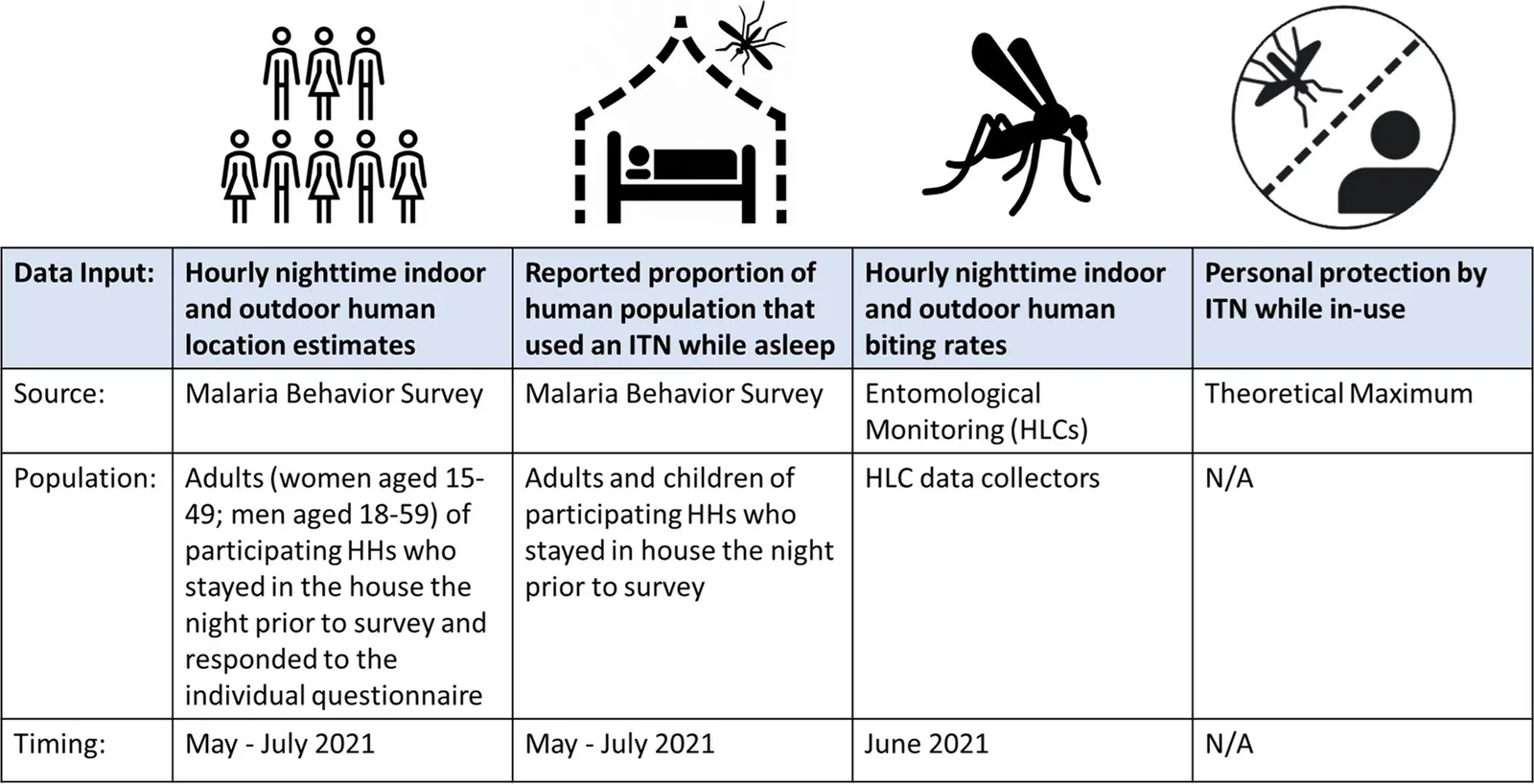
Source, population, and timing of respective inputs required for data integration

This case example integrated data from two separate existing sources, the MBS and routine VectorLink entomological monitoring, to estimate and characterize patterns of human exposure to malaria vectors in Central Malawi. The data sources as they relate to required inputs are summarized in Fig. 2. Further details about how these data were extracted from each data source are included in the following section.

### Vector behavior and location

Mosquitoes were collected at four sites/villages in three of nine districts within Malawi’s Central zone in June 2021: HLCs were conducted indoors and outdoors for extended collection hours from 5:00 PM to 11:00 AM [31, 38] to determine the biting rates during early evening and late morning hours, feeding locations (indoors and outdoors), and peak biting time of various *Anopheles* vectors [27]. Collection counts per hour were disaggregated by species: *Anopheles funestus* s.l. and *An. gambiae* s.l. HLCs were conducted for three nights at two houses per night (a total of six houses per site) using one collector indoors and one collector outdoors (approximately five meters from the house) Indoor and outdoor collectors switched places each hour to minimize collector bias, and the team of collectors were swapped with new collectors every six hours of collection to mitigate collector fatigue.

To calculate the indoor human biting rate (HBR), the total count of each mosquito species collected indoors for each hour was divided by the total number of human collectors indoors (six) per hour for each site. The same calculation was followed to calculate the outdoor HBR.

### Human behavior and location

Estimates of indoor and outdoor human location data and population level ITN access and use in central Malawi were derived from the Malawi MBS, a cross-sectional community-based survey with structured questionnaires administered to a random sample of women and men of reproductive age and heads of households. The Malawi MBS was fielded in May through July 2021 and was conducted nationwide in three survey zones of Malawi comprising the North, Central, and South regions. For this data integration, a set of data including ITN access, ITN use, and human location and sleep behavior of individuals within the central MBS zone were extracted for use, consisting of 1,302 households (for the household survey) and 432 men and 1346 women (for the individual survey) [35].

The following set of questions, answered by all respondents in the MBS individual questionnaires [36], was used to calculate human location estimates:

- Approximately at what time did you go to sleep yesterday?
- Approximately at what time did you wake up today?
- Did you sleep indoors or outdoors?
- What time did you go indoors for the evening?
- What time did you go outdoors for the morning?

For questions involving specific times, data collectors were instructed to round responses to the nearest whole hour, 0:00 to 23:00.

### ITN access and use

The proportion of the human population that used an ITN while asleep was calculated from the MBS using the same method as the MIS [37]: the percentage of the de facto household population (all individuals who stayed in the household the previous night) that used an ITN the night before the MBS [35]. This was determined from a net roster created by household survey respondents, which identified all adults and children who stayed in the household, all ITNs available within the household for use, and who stayed under which nets the night before the MBS. While not directly factored into indicators of human-vector interaction, ITN access and the use: access ratio – the ratio of individuals who have access to an ITN who reported using an ITN the previous night – were also calculated to better understand the extent to which gaps in use were driven by levels of access. To calculate the proportion of the population that had access to an ITN, the number of potential ITN users was calculated by multiplying the total number of nets in each household by two (assuming one net can serve two people). This number of potential users was divided by the de facto members for each household to determine the overall access by household. The overall ITN use was divided by overall ITN access to calculate the use given access ratio, representing the proportion of the population with access to a net that used it.

### Estimate of personal protection provided by ITNs while in use

Previous calculations of vector-human interactions have incorporated estimates of personal protection provided by ITNs while in use based on blood feeding inhibition shown in experimental hut trials [33, 34]. However, as many factors impact the personal protection of an ITN – including the ITN’s condition, age, and durability; human behavior and use of the ITN; and vector biting patterns and insecticide resistance profiles–estimates available in the literature did not reflect the situation and context from the time of the human and vector data collection. The subsequent vector-human interaction calculations are based on the maximum theoretical protection that an ITN could provide: 100% protection for an individual ITN user. This means that it was assumed a person was protected from all potential mosquito bites during the time they were under the protection of an ITN.

### Ethical approval

No new data were collected as part of this study. The MBS in Malawi received Institutional Review Board (IRB) approval from the Johns Hopkins School of Public Health IRB (IRB #15731) as well as the National Health Sciences Research Committee (NHSRC #19/11/2247) prior to survey data collection. All participants consented via written informed consent before survey administration. HLCs were approved by the National Malaria Control Program. Verbal consent was obtained from each household to conduct HLCs in their homes, and written consent was obtained from each HLC collector. Each HLC collector was given malaria chemoprophylaxis prior to collections.

### Results

Results are presented below, in the following categories: vector behavior, human behavior, and behavior-adjusted vector-human interactions.

### Vector behavior

**Fig. 3:**
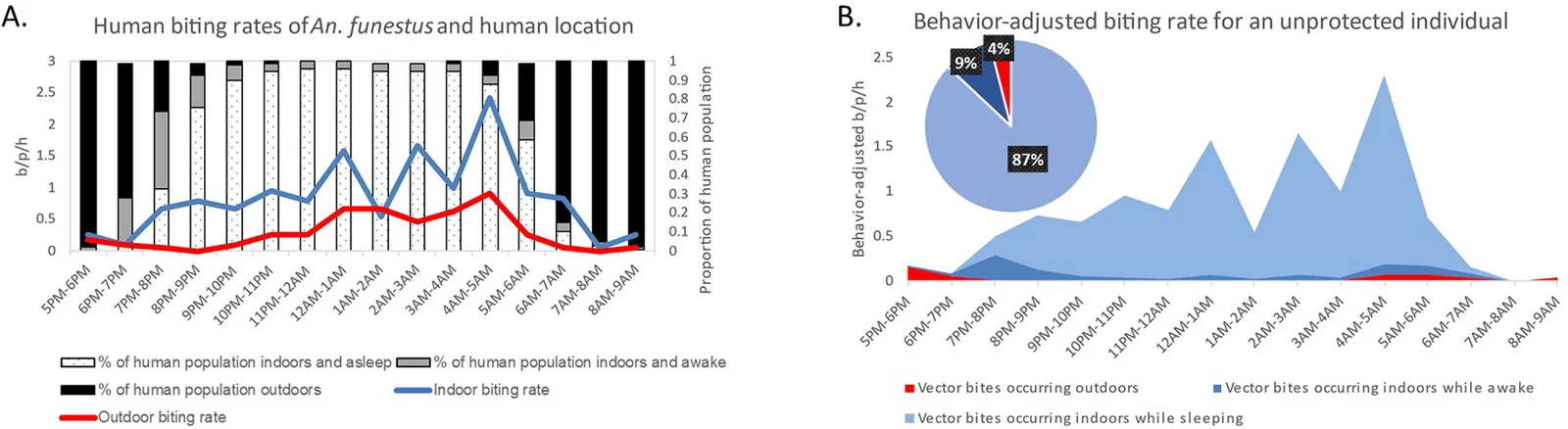
**A**–**B**. **A** Directly measured indoor and outdoor biting rate (measured in bites per person per hour, b/p/h) of *An. funestus* s.l., overlaid on the proportion of the human population indoors and asleep, indoors and awake, or outdoors. **B** Behavior-adjusted exposure of an unprotected individual to *An. funestus* s.l. Graph indicates behavior-adjusted bites per person per hour. Pie chart indicates the total percentage of exposure to vector bites estimated to occur within each category

A total of 453 *An. funestus* s.l. and *An. gambiae* s.l. mosquitoes were collected by HLC in the central MBS zone (*n* = 432, 21 respectively). Of the 432 *An. funestus* s.l. collected, 74.8% were collected indoors (*n* = 323) and 25.2% collected outdoors (*n* = 109). The indoor HBR for *An. funestus* s.l. exceeded 1.00 bites/person/hour (b/p/h) for the hours 12:00 AM–1:00 AM and 2:00–5:00 AM, and peaked at 2.42 b/p/h (95% confidence interval (CI) 1.84–3.12 b/p/h) from 4:00–5:00 AM. Similarly, the outdoor HBR for *An. funestus* s.l. also peaked at 0.92 b/p/h (95% CI 0.57–1.39) during the early morning hours from 4:00–5:00 AM (Fig. 3A). As hourly collection counts for *An. funestus* were less than 25 for each time point at each entomological surveillance site–thus limiting data interpretation individually–sites were consolidated by hour for analysis within the central MBS zone. To calculate the HBR for the central MBS zone, the total indoor *An. funestus* s.l. counts for 4 sites were averaged per hour and divided by the number of collectors indoors per hour [6]. Although HLCs were conducted from 5 PM to 11 AM, no mosquitoes were collected after 9 AM; all figures showing HBR by hour show the subsection of data from 5 PM to 9 AM, representing the period that mosquitoes were collected.

Only 21 *An. gambiae* s.l. were collected via HLCs over the same period described which, when disaggregated by hour and location, limited analysis. Given that so few *An. gambiae* s.l. were collected by HLC in June 2021 and vector bionomics vary between species, *An. gambiae* s.l. HBR was omitted and only HBR for *An. funestus* s.l. was carried forward for subsequent analyses.

### Human behavior

Overall, most respondents were outside from 5:00 PM to 7:00 PM. Between 7:00 and 8:00 PM, however, the majority of respondents (*n* = 1226, 74%, 95% CI 71–78%) moved indoors, with about half of these respondents indoors but still awake (*n* = 680, 41%, 95% CI 38–44%). After 8:00 PM, most respondents were indoors and asleep (8:00 PM–9:00 PM: *n* = 1260, 76%, 95% CI 73–79%); 9:00 PM–10:00 PM: *n* = 1492, 90, 95% CI 88–92%) until the following morning. Most exited the house by 6:00 AM (6:00 AM–7:00 AM: *n* = 1426, 86, 95% CI 83–88%) (Fig. 3A).

At the time when the MBS was conducted (May–July 2021), approximately 39% (*n* = 2241, 95% CI 35–42%) of the study population reported having access to an ITN, and approximately 40% (*n* = 2298, 95% CI 37–44%) of the same population reported using an ITN the night before the survey. This resulted in a use:access ratio of 1.03.

### Behavior-adjusted vector-human interaction and gaps in protection

Most mosquitoes were collected indoors (*n* = 323, 75%). Both indoor and outdoor biting rates peaked at night–after twilight, beginning around 7 PM in this context–and in the early morning, which coincides with the times that most people were reported to be indoors and asleep (Fig. 3A).

When accounting for where people spend time throughout the night, for an unprotected individual (i.e., someone who did not use an ITN at any point during the night), an estimated 96% of vector bites would occur indoors, with 87% of bites estimated to occur indoors while an individual was asleep (Fig. 3B). The remaining exposure to vector bites was estimated to occur outdoors (4%) and indoors while an individual was awake (9%) in the evening or early morning hours. Exposure for an unprotected individual was estimated to peak indoors, early in the morning, from 4:00–5:00 AM.

**Fig. 4:**
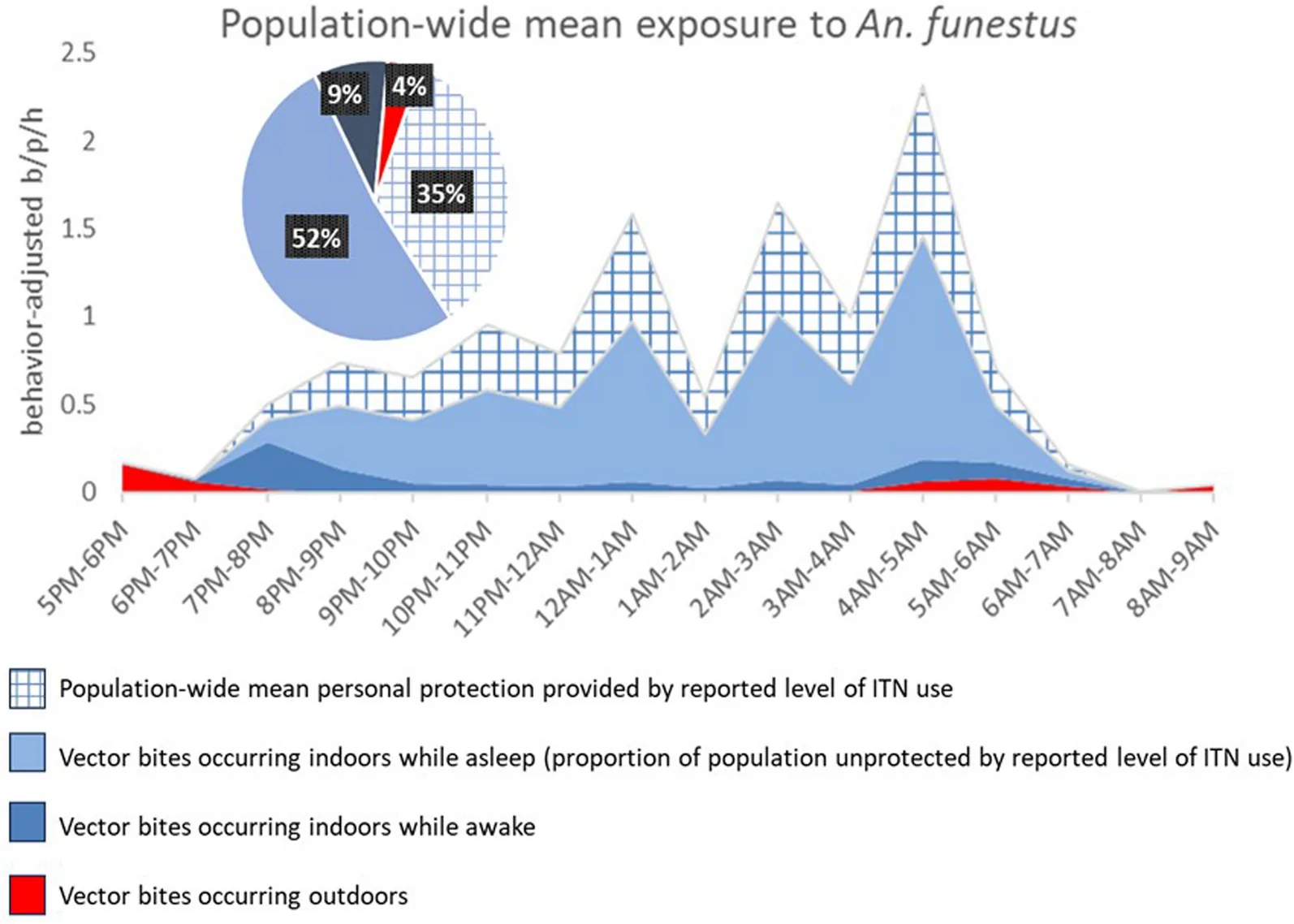
Estimated population-wide mean exposure to vector bites by hour, taking into account reported level of ITN use. Graph indicates behavior-adjusted bites per person per hour. Pie chart indicates the total percentage of exposure to vector bites estimated to occur within each category

An estimated 87% of vector bites occurred indoors during sleeping hours, representing the maximum percentage of bites that could be prevented by ITN use. When factoring in the percentage of the population that was estimated to use an ITN, the percentage of vector bites prevented by ITN use was 35% (Fig. 4).

## Discussion

### Interpretation and implications

While the results of the data integration are presented as point estimates, during interpretation, emphasis is placed on which exposures are of greater importance in relation to the others. The results of this data integration suggest that, in the central region of Malawi, most potential population-wide exposure to the malaria vector species *An. funestus* s.l. occurs indoors, during sleeping hours. The estimated percentage of potential exposure occurring indoors for an unprotected individual–representing the maximum possible personal protection that any indoor intervention could provide–was 96%, while the estimated percentage of potential exposure occurring while an unprotected individual was asleep–representing the maximum possible protection that any intervention targeting sleeping spaces could provide–was 87%. Importantly, although the estimates for ITN access and use in the study area were low (39% and 40%, respectively), the reported use *given* access was quite high (use:access ratio of 1.03), indicating that people were using the nets that they had. Given that most human exposure to vector bites was estimated to occur indoors and during times when people were asleep, high coverage of effective interventions that target sleeping spaces could greatly reduce human exposure to vectors. Given the strong culture of net use, if available, the data suggest ITNs could continue to be highly effective interventions in this context, with the potential to substantially reduce exposure to vector bites.

While these data indicate that ITNs may be well suited for the context of this study conducted in Central Malawi, the results may also be used to examine areas where gaps in protection remain. Such gaps may be either related to human behavior, vector control implementation quality or access, or intervention effectiveness. The high ITN use:access ratio suggests that there was not a behavioral gap in using ITNs in this context. Instead, there may have been a gap in protection related to ITN access with a lack of ITNs for high population coverage. When the data integration accounted for human location and sleeping times in relation to vector biting patterns, ITNs were estimated to prevent up to 87% of bites. However, when factoring in the level of ITN use in the population, the estimated proportion of exposure prevented was 35%. Access is likely a main driver in the gap seen between the maximum possible protection by an ITN and the protection estimated at the population level. To illustrate how ITN access–which was taken as an equivalent for use in this context, as most people used the nets they had access to–might affect the results, the target ITN use rate of 80% was modeled with the same methods (Additional File 2). This exercise suggested that, at 80% population ITN use, ITNs were estimated to prevent up to 70% of human exposure to vector bites, thus narrowing the gap between the maximum possible amount of exposure seen in the individual net use rates and what was seen at the population level.

Since a theoretical maximum for the personal protection provided by ITNs while in use was used in the calculations, other factors that were not directly included in the calculation, such as net durability, net condition, and insecticide resistance within the vector populations, should be taken into account when interpreting these results for decision making. The MBS, which estimated low access to ITNs (39%) in these areas of Malawi at the time these data were collected, also measured moderate to high rates of behaviors that are not recommended for ITN care and may have negatively impacted ITN condition and lifespan. Of all ITNs found in households in the Central Zone of Malawi, 30% were over-washed (reportedly washed more than twice in six months); 69% were washed with products that may damage the insecticide (i.e., powder or liquid soap); and 29% were dried in the sun [35]. Additionally, net durability monitoring data from 2019 indicates that ITN durability in this context showed ITN efficacy for two years, falling short of the expected three years, with high net attrition by 36 months post-distribution [38]. Previous studies also indicate that vector populations within Malawi have reduced susceptibility to insecticides commonly used in ITNs [39–41]. These factors would likely result in reduced effectiveness of ITNs while in use, as ITN condition and insecticidal activity would be less than that of the maximum theoretical protection used in the calculations [42]. With this in mind, programs in locations with an exposure profile similar to that identified in this study might seek to improve or maintain high ITN access, select the most effective ITN for the vector population, support communities to maintain the strong culture of ITN use (given access), and increase ITN care practices.

The identified gap in protection around ITN access is in contrast to gaps due to outdoor transmission or bites occurring outside of sleeping hours, which, due to the scaling-up of IRS and ITNs over the past decades, are often identified as main drivers of residual malaria transmission in other contexts [18, 24, 33, 43–45]. However, the results from this study align with a review examining patterns of vector-human exposure, which found that most exposure occurred indoors during sleeping hours for non-users of ITNs [20].

The NMCP has been implementing malaria vector control measures prior to and after the period during which this data was collected. From 2021 to 2023, the NMCP conducted targeted mass distributions of piperonyl-butoxide (PBO) and dual active ingredient (AI) ITNs to 25 districts where IRS was not being implemented, to mitigate insecticide resistance. Prior to the 2021 distribution, standard ITNs were used during the mass campaign. In 2024, a mass ITN distribution targeted all 29 districts, with a distribution of dual AI ITNs (IG2 and PermaNet dual). Given the reduced durability of ITNs in Malawi–from an expected three years to only two years–the country has changed its policy to conduct mass net distributions every two years. The country is currently seeking donor support to implement this updated policy. Additionally, the NMCP has intensified social and behavior change activities to promote proper ITN use and care at the community level. As part of these efforts, the country launched the Malaria Youth Corps, a youth-led civil society organization that mobilizes the communities on malaria control, in October 2024. Additionally, the PMI VectorLink Malawi project implemented IRS (Actellic 300CS and/or SumiShield 50WG) in Nkhotakota district from 2018 through 2023, adding three additional districts (Balaka, Mangochi, and Nkhata Bay) in 2020 with added support from World Vision International (funded by Global Fund). In all other districts that were not covered by IRS, the NMCP distributed a mixture of PBO and dual AI ITNs were distributed via mass distribution campaign by the NMCP from 2021 (after the time that data was collected for the present analysis) to 2023 [46].

### Limitations and considerations for interpretation

This study is subject to a number of limitations. First, we assumed the level of personal protection provided by ITNs in use as 100%, or the theoretical maximum protection. Personal protection from experimental hut studies generally varies between 50 and 90%, with some nets providing near-complete protection in ideal conditions. Personal protection is likely affected by the type of ITN and insecticide on the nets, the susceptibility status of the local mosquito population, and the quality of the ITN, including insecticide concentrations on the nets and physical condition. In interpreting the results, the overall patterns of exposure should be used to inform the gaps in protection, and additional sources–described herein–will provide important contextualization to the interpretations. Net durability monitoring reports can be a key resource in understanding ITN longevity and therefore provide context to estimates of ITN access and effectiveness in the time period following a distribution. Information on insecticide resistance of the malaria vectors in the study area can help interpret the calculated human exposure patterns. Additional data on human behavior from outside of the peri-domestic space can provide insights into remaining gaps in exposure and protection during time that people spend away from home, either during brief periods when they leave the house at night (e.g., to use the restroom) or for community-wide events (e.g., to attend funerals, festivals, markets) [20, 47–49]. The estimate of personal protection also did not factor in the potential protection conferred by the community effect on malaria transmission by ITNs as high coverage of ITNs has been shown to reduce the densities and longevity of mosquito populations [50–53].

Second, SumiShield 50WG, which was used for IRS, remained highly effective in the context for up to 8–10 months post-spray [27, 54], and the entomological monitoring for this study was conducted within this window of the most recent IRS campaign. As access was estimated through the MBS, which is generalizable at the Central zone as a unit, the ITN access rates may have been lower due to Nkhotakota not being targeted for mass ITN campaigns due to IRS. Spraying also likely contributed to lower numbers of vectors collected overall. While the absolute numbers were low, there were sufficient numbers of *An. funestus* s.l. to look at patterns of exposure. Additionally, while there were few mosquitoes collected through HLCs in this region that were not *An. funestus* s.l.*,* the calculations in this study focused only on this one major vector species; exposure patterns may differ with other species.

Third, while the dates of human and vector behavior data collection overlapped in 2021, the data for ITN access rates were collected almost three years after the most recent mass distribution of ITNs in 2018. As durability monitoring studies indicate that ITNs may last less than three years in some contexts [42–44], degrading physical and chemical net composition over nearly three years likely impacted ITN access, and in turn use, in the study area. Indeed, other studies, with timing 1–2 years from the 2018 mass distribution campaign, estimated ITN access to be near 50% [55, 56] and ITN target access rates following mass distribution are 80%, as compared to the estimated ITN access near 40% at the time of this study. Relatedly, the case example examined vector-human exposure retrospectively, integrating data that were readily available but collected over two years in the past. Due to the cross-sectional nature of the MBS, this analysis was also unable to account for interannual or seasonal variability in either vector or human behavior. Other historical data points may provide additional context around how human behavior and ITN access and use fluctuate over time–both seasonally and annually–in the particular context.

Lastly, while the MBS provided a sizable data sample, it was powered to be generalizable at the level of the Central zone as a whole. Conversely, HLC data were only available from four locations within the Central zone. Recognizing that vector behavior can be variable even in geographically similar areas, these differences may have been minimized by averaging vector behavior data across sites, even in the same region, and the HLC data may not have been representative of the Central zone’s vector population as a whole. A study from Bioko Island in Equatorial Guinea emphasized heterogeneous vector behavior may be even within a given geography and how these variances translate to differences in exposure patterns [18]. Additionally, while the ITN access and use estimates include both adult and child behaviors, the hourly nighttime human behavior estimates were derived from a questionnaire conducted only with adult men and women. Complementing this analysis with additional human behavior data from a more granular level, like those collected through VectorLink sentinel sites [54, 57] or specific to children’s behaviors, may allow for finer-scale triangulation or age stratification of results, although small sample sizes will need to be interpreted carefully. Additionally, more complex modeling may incorporate temporal trends to further refine results. These limitations were taken into consideration while interpreting outcomes from the vector-human data integration and emphasize the need to interpret these results as patterns of exposure to identify which gaps in protection may be more pressing relative to others, rather than absolute values.

## Conclusion

Identifying when and where gaps in protection against malaria vector bites occur is crucial for successful interventions, making it essential to use local data for this purpose and continuously monitor access to ensure planned distribution channels are effective. Importantly, the value of coordination and collaboration among stakeholders–bringing together programs that specialize in entomology and human behavior, especially those within the country of implementation–should not be understated. Engaging this group was also key to interpreting outcomes of the data integration into constructive programmatic recommendations, as well as identifying missing elements in interpretation. Results suggest that ITNs were well accepted and used within Central Malawi, and that gaps in protection may be closed by increasing and maintaining high access to ITNs.

However, as this case example was retrospective, future studies should consider planning data collection for human and vector behavior elements prior to major vector control product procurements or national social and behavior change activities to provide of-the-moment gap identification in support of programmatic decision making. Additionally, further consideration should be given to understand the extent to which the data elements can be generalized, geographically and temporally, as well as the intrinsic limitations of these estimates when approximating real world conditions. Overall, this case example suggests that integrating routinely collected entomological data with a standard set of human and vector behavioral data elements can provide valuable information on potential gaps in protection to inform decision making.

## Supplementary Information

Supplementary Material 1

Supplementary Material 2

## Data Availability

The datasets analyzed during the current study are available from the corresponding author on reasonable request.

## Abbreviations

AI: Active ingredient
ANC: Antenatal care
b/p/h: Bites/person/hour
CI: Confidence interval
HBR: Human biting rate
HLC: Human landing catch
IRB: Institutional Review Board
IRS: Indoor residual spraying
ITN: Insecticide-treated bed net
MAC: Malaria Alert Centre
MBS: Malaria Behavior Survey
MIS: Malaria Indicator Survey
NHSRC: National Health Sciences Research Committee
NMCP: National Malaria Control Program
PBO: Piperonyl butoxide
